# Things Old and New

**Published:** 1871-04

**Authors:** W. C. Stanley


### THINGS OLD AND NEW.

BY W. C. STANLEY.

On the 17th of February last a young man, aged 18 years, who was suffering severe pain in the region of the lower incisor teeth, called upon me. On examination I found no decay, but the nerve in the left central incisor was dead, and as a result abscess was in progress. This in connection with the fact that the crown was somewhat misshapen, its axis also having a different inclination from the other incisors, and that it was almost crowded out of the arch, determined me to extract it. On doing so I found it to be a malformation, which I do not recollect to have seen mentioned in any dental work. I am sure that neither Tomes nor Harris speak of it. It is a tooth formed by the union of the crown of the temporary left central inferior incisor with the root and a portion of the crown of the permanent tooth of the same place. A temporary crown on a permanent root.

To get an idea of it, take a temporary and permanent tooth for that place, cut off half the permanent crown by a transverse section; this will leave a narrow belt of thin enamel on the mesial and distal side, but it will extend up considerably on the labial and palatal sides.

Now ream out the dentine and push the tapering root of the temporary tooth into it till it reaches the neck, and you have the thing complete.

The enamel of the partial permanent crown is perfectly fused around the neck of the temporary tooth, and yet it terminates on the labial and palatal sides as abruptly as if cut off with a file. The two structures are entirely distinct, the temporary crown being quite yellow, while the enamel of the partial permanent crown is pearly white and corresponds with that of the other teeth. The root is not completed, the foramen being large, oval, and with its edges sharp.

